# Comparison of the Effect of Intravitreal Dexamethasone Implant in Vitrectomized and Nonvitrectomized Eyes for the Treatment of Diabetic Macular Edema

**DOI:** 10.1155/2018/1757494

**Published:** 2018-04-22

**Authors:** Sadık Görkem Çevik, Sami Yılmaz, Mediha Tok Çevik, Fatma Düriye Akalp, Remzi Avcı

**Affiliations:** ^1^Department of Ophthalmology, Yüksek Ihtisas Research and Training Hospital, Bursa, Turkey; ^2^Retina Eye Hospital, Bursa, Turkey; ^3^Sisli Hamidiye Etfal Research and Training Hospital, İstanbul, Turkey; ^4^Department of Ophthalmology, Acıbadem Hospital, Bursa, Turkey

## Abstract

**Purpose:**

To compare the effectiveness of sustained-release dexamethasone (DEX) intravitreal implant in nonvitrectomized eyes and vitrectomized eyes with diabetic macular edema (DME).

**Methods:**

A retrospective review of the medical records of 40 eyes of 30 consecutive patients with diabetic macular edema who underwent intravitreal DEX implant injection. Patients were divided into 2 subgroups: 31 eyes that were nonvitrectomized (group 1) and 9 eyes that had previously undergone standard pars plana vitrectomy (group 2). The main outcome measures were BCVA and foveal thickness (FT).

**Results:**

A significant improvement was seen in BCVA in both group 1 and group 2 at the 1st, 2nd, and 6th months after treatment with DEX implant (*p* < 0.05). In group 1, a significant reduction in FT was observed at the 1st, 2nd, and 6th months (*p* < 0.05). In group 2, a significant reduction in FT was seen at the 1st and 2nd months (*p* < 0.05), but the reduction rate at the 6th month after the injection was not statistically significant (*p* = 0.06).

**Conclusion:**

DEX implant is effective for the treatment of diabetic macular edema, and the effectiveness of the drug is similar in vitrectomized and nonvitrectomized eyes.

## 1. Introduction

Diabetic macular edema is one of the most important causes of blindness worldwide particularly affecting those individuals of working age [1–3]. Since the recognition of the role of inflammation and importance of the vascular endothelial growth factor (VEGF) in the pathogenesis of diabetic retinopathy, treatment options have been altered with anti-VEGF drugs, and corticosteroids have taken an active role in the treatment of diabetic retinopathy [4–7]. Dexamethasone and triamcinolone are the most frequently used corticosteroids. Although dexamethasone is powerful, it remains in the vitreous for a limited amount of time; therefore, attempts have been made to support it with carriers which can remain longer in the vitreous [8, 9].

Despite the many new treatments that are available, a pars plana vitrectomy (PPV) is still required in some diabetic patients. The vitreoretinal tractions can be released, and the inflammatory stimulators that cause macular edema can be removed via PPV [10]. By its very nature, repeated intravitreal injections may be required in post-PPV patients. Anti-VEGF drugs, 5-FU, triamcinolone, and amphotericin B have been observed to clear out more rapidly in patients who have undergone PPV; thus, the effective duration has been reported to be shorter than expected [11–15].

The positive effects of corticosteroids in cases of diabetic macular edema have been known for many years. The effect of corticosteroids is achieved through the lowering of ICAM-1 gene expression and the VEGF level [17–19]. To prolong the corticosteroid half-life, carrier platforms which dissolve within the vitreous over a longer period of time have been developed, with Ozurdex being a significant long-term release dexamethasone implant. Ozurdex contains 0.7 mg of dexamethasone within its carrying system (Novadur® Styrolution; Aurora, Illinois, USA). It dissolves into lactic and glycolic acid in the vitreous, and this dissolution generates a slow dexamethasone release [20–22]. Following the implantation, an intense effect occurs during the first 2 months, which continues for 6 months [23].

This research aimed to compare the effective duration of a slow-release dexamethasone implant (Ozurdex) in vitrectomized and nonvitrectomized eyes for the treatment of diabetic macular edema.

## 2. Materials and Methods

This study was conducted in accordance with the Declaration of Helsinki, and informed consent forms were obtained from all of the patients. Our study involved a retrospective review of 40 eyes of 30 patients who were treated with Ozurdex implant injections for diabetic macular edema under operating room conditions at the Bursa Retina Eye Hospital between January 2012 and May 2015. Each intravitreal implant was applied under topical anesthesia with a 22 G applicator, 3.5 mm posterior to the limbus. The patients were divided into two: group 1 (*n* = 31), patients who were not operated on, and group 2 (*n* = 9), patients who had undergone vitrectomy surgery. The patients in group 1 had diabetic retinopathy, and those patients were previously treated with panretinal laser photocoagulation, intravitreal anti-VEGF (bevacizumab), or triamcinolone injections. Combined vitrectomy surgeries (phacoemulsification + PPV + endolaser treatment + gas tamponade + ILM peeling) were performed due to complications of diabetic retinopathy in all of the group 2 patients.

The age, gender, lens status of the patients, and previous treatments like focal-grid laser or other intravitreal therapies like anti-VEGF and triamcinolone were recorded. We used bevacizumab as anti-VEGF drug, and we did not switch throughout the study period. The inclusion criteria were the presence of intraretinal fluid demonstrated by optical coherence tomography (OCT) and at least 60 days since the last treatment. The exclusion criteria were a history of glaucoma, any kind of vitreomacular traction, uncontrolled diabetes (HbA1c > 8), incomplete laser treatment, and cases which required vitreous surgery during the follow-up. Recurrence of DME was defined as new intraretinal fluid showed by OCT after the final injection.

For each patient, the best-corrected visual acuity (BCVA) (Snellen converted logMAR) and macular thickness on OCT at baseline visit and within the 1st, 2nd, 6th months, and during follow-up visits were obtained. The OCT measurements were taken with a spectral domain OCT device (Heidelberg, Germany). Those patients who presented with complaints of visual impairment were measured for visual acuity and macular thickness. The changes in the BCVA, the presence of intraretinal fluid and intraocular pressure (IOP), the complications, and the time to recurrence were evaluated. When recurrence of DME was observed, a second injection was performed after the 6th month.

The statistical analyses were performed using IBM SPSS 22.0 statistics software. The Wilcoxon signed-rank test was used for the determination of the visual acuity and the changes in foveal thickness in the groups. The Chi-squared test was used in the evaluation of the distribution of the patients with recurrence, while the Mann–Whitney *U* test was used in the evaluation of the time to recurrence. A value of *p* < 0.05 was accepted as statistically significant.

## 3. Results

The mean age of patients was 63.1 ± 8.1 (47–85) years in group 1 and 60.4 ± 9.2 (47–77) years in group 2. The mean follow-up time was 19.7 ± 11.2 (6–36) months in group 1 and 15.8 ± 9.3 (6–36) months in group 2. Focal laser photocoagulation treatment was performed before the intravitreal Ozurdex therapy in 25 eyes (71%) in group 1 and in 9 eyes (100%) in group 2. The intravitreal steroid (except Ozurdex) and/or anti-VEGF (bevacizumab) injections were given before the intravitreal Ozurdex therapy in 27 eyes (87%) in group 1 and in 8 eyes (88%) in group 2. Fourteen eyes (45%) in group 1 and 9 eyes (100%) in group 2 had pseudophakia (Table 1).

In group 1, the best-corrected visual acuity (BCVA) was 0.88 ± 0.46 at the baseline visit, 0.60 ± 0.38 at the 1st month, 0.56 ± 0.32 at the 2nd month, and 0.58 ± 0.41 at the 6th month (logMAR). In group 2, the BCVA was 0.98 ± 0.66 at the baseline visit, 0.67 ± 0.48 at the 1st month, 0.54 ± 0.26 at the 2nd month, and 0.77 ± 0.58 at the 6th month (logMAR). When compared to the baseline values, the increases in the BCVA in group 1 within the 1st, 2nd, and 6th months were statistically significant (*p* < 0.001, *p* < 0.001, and *p* < 0.001, resp.). In group 2, the increases in the BCVA were also statistically significant at the 1st, 2nd, and 6th months when compared to the baseline levels (*p* = 0.011, *p* < 0.001, and *p* = 0.048, resp.).

In group 1, the macular thickness was 596 ± 170 (265–1004) microns prior to injection, 403 ± 108 (160–575) microns at the 1st, 304 ± 74 (190–440) microns at the 2nd, and 339 ± 176 (88–835) microns at the 6th month. In group 1, the changes in the macular thickness were statistically significant at the 1st, 2nd, and 6th months when compared to the baseline visit values (*p* < 0.05). The macular thickness in group 2 at baseline visit was 547 ± 166 (354–810) microns, and it was 358 ± 99 (200–505) microns at the 1st, 221 ± 105 (150–350) microns at the 2nd, and 349 ± 178 (164–700) microns at the 6th month. The changes in the macular thickness within the 1st and 2nd months in group 2 were statistically significant (*p* = 0.012 and *p* < 0.011, resp.) when compared to the baseline values, but the change at the 6th month was not statistically significant (*p* = 0.06) (Table 2).

During the first 6-month period, the time to recurrence of macular edema was 20.2 ± 2.2 weeks in group 1 and 17.5 ± 4.4 weeks in group 2, but there was no statistically significant difference between the 2 groups (*p* = 0.082). Recurrences were observed in 16 eyes in group 1 and in 4 eyes in group 2, but there was no statistically significant difference between the 2 groups (*p* = 0.076).

High IOP (HIP) values, which can be brought under control with medical treatment, were measured as >25 mmHg in 2 eyes in group 1 and in 1 eye in group 2, although none of these measurements exceeded 30 mmHg. Within the first 6-month period, cataract development was observed in 2 eyes in group 1. Moreover, all of the group 2 patients had pseudophakia. Cataract progression was clinically observed. Visual acuities of the cases with cataract were removed from statistical analysis.

Of the patients with recurrence, a second dexamethasone implant was applied to 8 of the patients who accepted treatment in group 1 and to 2 of the patients in group 2. The remaining patients with recurrence did not accept another intravitreal injection. Intravitreal anti-VEGF (bevacizumab) treatments were applied to 2 patients following recurrence in group 1 because of the HIP. The recurrences of diabetic macular edema were observed in 6 eyes that had undergone second dexamethasone implant injections in group 1. In those eyes, the mean recurrence time was 21 ± 2.6 (19–25) weeks. We observed a recurrence of diabetic macular edema during the 21st week in 1 patient who had undergone a second intravitreal injection of a dexamethasone implant in group 2. A third dexamethasone implant was injected into 2 patients in group 1. Following repeated Ozurdex injections, the time to recurrence of DME extended from 21 ± 2.6 weeks to 25 ± 1.3 weeks (Table 3).

When we looked at the final results of those patients without recurrence during the first 6 months of follow-up, we saw recurrence in both group1 and group 2 during the following 6 months. In group 1, recurrence was observed in 8 of 15 eyes within 6 to 9 months of follow-up and in 5 eyes within 9 to 12 months. No recurrence was observed in 2 eyes in 36 months of total follow-up. Of those 9 eyes (out of 13) in which recurrence was observed after 6 months, 6 of them were treated with Ozurdex and 3 of them were treated with anti-VEGF (bevacizumab). Four eyes left untreated due to the patients' disapproval. In group 2, recurrence was observed in 4 eyes after 6 months, which were retreated with Ozurdex. No recurrence was observed within 36 months in only 1 eye.

## 4. Discussion

Following PPV surgery, frequently repeated application of intravitreal medication may be required; therefore, it is important to know how these medications will act on the eyes which have undergone vitrectomy surgery [16]. The results of this study showed that a dexamethasone implant was effective in both vitrectomized and nonvitrectomized eyes. After the removal of the vitreous, the eye becomes less viscous; therefore, a clearance of intravitreal drugs from the vitreus cavity is accelerated [11, 24, 25]. It is well known that the clearance of triamcinolone particles is much faster in vitrectomized eyes [26, 27]. Previous studies have shown a shorter half-life of anti-VEGF drugs in the eyes which have undergone PPV, and a 60% decrease has been observed in the half-life of bevacizumab in monkey eyes following PPV [28]. In a study by Niwa et al., comparing the half-lives of ranibizumab and aflibercept in vitrectomized and nonvitrectomized eyes, the half-lives of both molecules were decreased in vitrectomized eyes [29]. Moisseiev et al. calculated the half-life of bevacizumab on vitrectomized and nonvitrectomized eyes and reported that the half-life was 4.9 days in the nonvitrectomized eyes and 0.66 days in the vitrectomized eyes [30]. However, the Diabetic Retinopathy Clinical Research Network trial had shown favorable responses to intravitreal ranibizumab treatment in the management of DME among patients with prior vitrectomy [43].

In the current study, the increases in the BCVA during the 1st, 2nd, and 6th months, when compared to the baseline levels, were statistically significant in the nonvitrectomized and vitrectomized eyes, and all of the medications were seen to have similar effects in both groups. In a study comprised of 24 vitrectomized and 34 nonvitrectomized eyes by Dutra Medeiros et al., statistically significantly increases were observed in BCVA during the 1st, 3rd, and 6th months after the intravitreal Ozurdex injection. In the same study, statistically significant improvements in the foveal thicknesses of both the vitrectomized and nonvitrectomized eyes were observed during the 1st, 3rd, and 6th months when compared with the baseline levels [31]. Although we have found similar results in our study, the change in macular thickness in vitrectomized eyes at 6 months was not statistically significant. This nonstatistically significant result was attributed to the lower number of patients in group 2 (*p* = 0.06), which was a significant drawback of our study. Given the small number of cases in group 2, this condition may be interpreted as clinically significant.

The detection time of recurrent macular edema was an average of 17.5 weeks in the vitrectomized eyes and 20 weeks in the nonvitrectomized eyes, and this period was determined to be similar between the 2 groups. In a study by Escobar-Barranco et al., Ozurdex implants were applied to refractory and treatment naïve patients with diffuse diabetic macular edema, and the median time for reinjection was 4 months [32]. Hattenbach et al. studied 24 patients (25 eyes) who had undergone PPV and had persistent postoperative macular edema. The macular thickness decreased within 4 to 8 weeks after the Ozurdex implantation, and the visual improvement was observed in 79% of the patients. In the same study, the macular thickness was observed to increase between 10 and 16 weeks, and the dexamethasone implant was reapplied to 12 of 25 eyes [33]. In another study by Hattenbach et al., the medical records of 37 patients (39 eyes) who had been treated with intravitreal DEX implant for postoperative persistent cystoid macular edema following vitrectomy and peeling of idiopathic epiretinal membranes were retrospectively reviewed [39]. And they reported that 17 of 39 eyes (43.6%) necessitated minimum 1 repeat injection of DEX implant. Klamann et al. reported that intravitreal DEX implant is an effective treatment among patients with postoperative macular edema after cataract surgery or pars plana vitrectomy. They observed recurrence of macular edema in 8 patients 8.1 ± 5.3 months after the first injection of DEX which responded to reinjection [40]. In an experimental study, the behavior of Ozurdex implants injected into vitrectomized eyes was modeled, and Ozurdex was injected into a BSS-filled box at different release angles (15°, 30°, and 45°) [41]. They found that the implant injected at 15° reached the highest mean initial velocity and mean initial normalized energy. The combination of intravitreal DEX injection with pars plana vitrectomy had been shown to be safe and effective for some underlying conditions that result in macular edema, like diabetic retinopathy, retinal vein occlusion, and uveitis [42].

The results obtained in this study showed no statistically significant difference regarding recurrence of macular edema time between vitrectomized and nonvitrectomized eyes. The time to recurrence of macular edema was 20.2 ± 2.2 weeks in nonvitrectomized group and 17.5 ± 4.4 weeks in vitrectomized group.

Shah et al. evaluated the efficacy of intravitreal dexamethasone implants among 8 vitrectomized eyes with persistent diabetic macular edema, which were previously treated with intravitreal anti-VEGF therapy [37]. They observed an increase in the visual acuity and a decrease in the macular thickness 1 month after treatment. Additionally, that effect lasted for at least 3 months. Thanos et al. injected intravitreal Ozurdex implant to patients who had recalcitrant macular edema after a successful rhegmatogenous retinal detachment repair surgery. They observed recurrence of the edema in all patients at the end of 3 months [38]. In that study, the average number of implants to treat macular edema in their set of patients was 4 (range 1–14). In our study, we observed longer duration of the effect. Also, the second injection had greater efficacy than the first.

In a study by the Ozurdex CHAMPLAIN study group, a 26-week evaluation of the safety and efficacy of Ozurdex was conducted on 55 vitrectomized eyes via OCT scanning and foveal thickness examination. The findings showed a decrease in the maximum foveal thickness and an increase in vision in the 8th week, and these effects continued for 26 weeks. In the current study, the maximum increases in vision and decreases in the foveal thickness were observed in both patient groups in the postoperative 1st and 2nd months. The most frequently seen side effects in the Ozurdex CHAMPLAIN study group were conjunctival bleeding, an increase in the IOP, and pain; however, the medication was deemed to be safe [34]. In the current study, an increase in the IOP possibly requiring medical treatment was observed in 1 vitrectomized eye and 2 nonvitrectomized eyes. When the side effect profile of Ozurdex was evaluated, the increase in the IOP and development of cataract became prominent.

In the BEVORDEX study in which Ozurdex and bevacizumab were compared for 12 months, the IOP levels of 26% of the patients in the Ozurdex group were ≥25 mmHg, but they were lowered with medical treatment [35]. In the current study, all of the HIP cases were brought under control with medical treatment. In addition, cataract development is an important side effect of steroids that are applied intraocularly. In the MEAD study, in which dosages of 0.7 mg and 0.35 mg were compared, cataract development was determined at the rates of 67.9%, 64.1%, and 20.4%, respectively, for the 3 groups (including a 3rd control group) over a 3-year period. The rates of cataract surgery were 59.2%, 52.3%, and 7.2%, respectively, while in the dexamethasone implant group, cataract surgery was performed between 18 and 30 months in 75% of the patients [36]. In the current study, cataract development was observed in 2 eyes within the first 6-month period, with an expected increase at the longer term follow-up visits.

Our study had certain limitations, such as its retrospective nature, the small sample of eyes in group 2, and that the refusal of reinjection by some patients with recurrent macular edema. However, our follow-up time is long enough to evaluate the long-term effectiveness and safety of Ozurdex. In addition, we observed the period of recurrence extended as the number of injections increased in vitrectomized and nonvitrectomized eyes.

## 5. Conclusion

Overall, the sustained-release dexamethasone implant was shown to be similarly effective in both vitrectomized and nonvitrectomized eyes. When the duration of the effect on vitrectomized eyes in particular is taken into consideration, sooner, reinjection may be required in vitrectomized eyes to achieve the same results.

## Figures and Tables

**Table 1:** Results of the current study.

Eye	Patient age	Focal laser treatment	Intravitreal injection	Preoperative lens status	Complication	Vitrectomy surgery	Usage of treatment after recurrence (patients who accepted the treatment)
1	53	N	7	N	Ø	N	Dex
2	55	N	2	P	Ø	N	Dex
3	60	Y	4	N	Ø	N	
4	68	Y	2	N	Ø	N	
5	55	Y	4	P	Ø	N	Dex
6	55	Y	Ø	P	Ø	N	
7	68	Y	1	P	Ø	N	
8	68	Y	1	P	Ø	N	
9	77	Y	1	P	Ø	N	
10	67	Y	1	P	Ø	N	
11	67	Y	3	N	Ø	N	Dex
12	67	Y	3	N	Ø	N	Dex
13	67	Y	3	N	Ø	N	
14	85	Y	3	P	Ocular HT	N	Anti-VEGF
15	52	Y	1	N	Ø	N	
16	69	Y	7	N	Cataract	N	
17	71	N	2	P	Ø	N	
18	54	N	1	P	Ø	N	
19	54	Y	1	N	Ø	N	
20	60	Y	1	N	Ø	N	
21	77	Y	1	P	Ø	N	
22	58	N	2	N	Ocular HT	N	Anti-VEGF
23	47	Y	1	N	Ø	N	
24	65	Y	1	N	Ø	N	Dex
25	65	Y	2	N	Cataract	N	Dex
26	60	Y	2	N	Ø	N	Dex
27	67	Y	1	N	Ø	N	
28	57	Y	1	N	Ø	N	
29	65	N	Ø	P	Ø	N	
30	63	Y	Ø	P	Ø	N	
31	63	Y	Ø	P	Ø	N	
32	55	Y	1	P	Ø	Y	
33	51	Y	1	P	Ø	Y	
34	47	Y	1	P	Ø	Y	Dex
35	77	Y	2	P	Ø	Y	Dex
36	65	Y	1	P	Ø	Y	
37	60	Y	Ø	P	Ø	Y	
38	60	Y	1	P	Ø	Y	
39	59	Y	2	P	Ø	Y	
40	70	Y	3	P	Glaucoma	Y	

Y: yes; N: no; P: pseudophakic; N: natural; Dex: dexamethasone implant.

**Table 2:** Results of the current study.

Eye	Prein. VA (Snellen)	Postin. 1st month VA (Snellen)	Postin. 2nd month VA (Snellen)	Postin. 6th month VA (Snellen)	Prein. foveal thickness (*μ*)	Postin. 1st month foveal thickness (*μ*)	Postin. 2nd month foveal thickness (*μ*)	Postin. 6th month foveal thickness (*μ*)	Time to recurrence (week)	Group
1	20/2000	20/800	20/400	20/2000	752	575	300	380	16	1
2	20/1600	20/400	20/400	20/1600	550	368	280	471	20	1
3	20/100	20/80	20/80	20/40	577	270	244	200	Ø	1
4	20/100	20/100	20/100	20/100	454	438	350	476	22	1
5	20/200	20/100	20/100	20/125	662	291	190	835	19	1
6	20/40	20/32	Ø	20/25	350	242	200	240	Ø	1
7	20/40	20/40	Ø	20/25	383	364	Ø	300	Ø	1
8	20/400	20/400	Ø	20/200	853	563	Ø	104	Ø	1
9	20/200	20/100	Ø	20/125	413	400	Ø	163	Ø	1
10	20/100	20/50	Ø	20/40	642	400	Ø	485	20	1
11	20/40	20/40	20/40	20/125	662	540	265	481	24	1
12	20/40	20/32	20/32	20/63	550	432	380	463	20	1
13	20/200	20/125	20/125	20/125	1004	560	390	204	Ø	1
14	20/2000	20/400	20/400	20/200	330	200	200	250	22	1
15	20/125	20/40	20/40	20/80	607	400	305	334	Ø	1
16	20/125	20/40	20/40	20/80	529	356	290	200	Ø	1
17	20/50	20/32	20/32	20/25	525	390	280	173	Ø	1
18	20/125	20/100	Ø	20/40	496	337	Ø	490	20	1
19	20/400	20/125	Ø	20/63	884	540	Ø	150	Ø	1
20	20/200	20/50	20/50	20/40	826	550	350	88	Ø	1
21	20/200	20/125	20/200	20/200	525	332	300	118	Ø	1
22	20/50	20/40	20/40	20/63	716	505	440	553	16	1
23	20/200	20/125	20/100	20/200	808	510	430	162	Ø	1
24	20/63	20/40	20/125	20/40	741	450	400	450	21	1
25	20/200	20/125	20/125	20/63	592	480	190	363	20	1
26	20/200	20/63	20/63	20/50	516	440	320	419	24	1
27	20/200	20/100	Ø	20/125	479	400	Ø	186	Ø	1
28	20/200	20/63	Ø	20/63	265	160	Ø	302	20	1
29	20/40	20/40	20/63	20/25	620	325	300	600	20	1
30	20/40	Ø	Ø	20/25	590	364	Ø	317	Ø	1
31	20/125	Ø	Ø	20/100	598	335	300	560	20	1
32	20/200	20/100	20/100	20/125	810	387	350	283	Ø	2
33	20/40	Ø	20/32	20/32	463	Ø	Ø	358	Ø	2
34	20/50	20/40	20/40	20/40	449	250	210	700	16	2
35	20/200	20/100	20/50	20/100	708	505	310	468	22	2
36	20/200	20/50	20/50	20/125	768	450	150	164	Ø	2
37	20/200	20/50	20/40	20/50	456	375	180	165	Ø	2
38	20/2000	20/500	20/400	20/500	457	374	200	166	Ø	2
39	20/2000	20/500	20/500	20/2000	458	323	180	389	20	2
40	20/32	20/25	20/32	20/50	354	200	190	450	12	2

**Table 3:** Recurrence and retreatment number and times.

	Total	Retreatment	Avg. recurrence time	Second retreatment	Secondary recurrence	Avg. recurrence time 2	Retreatment
Recurrence in 6 months							
Group 1	16 eyes	8 (out of 16)	21 ± 2.6 weeks	8 eyes	6 eyes	25 ± 1.3 weeks	2 eyes
Group 2	4 eyes	2 eyes	21 weeks	1 eye			
Recurrence > 6 month							
	6–12 months						
Group 1	20 eyes						
Group 2	6 eyes						
Recurrence > 6 month							
Group 1	20	9	24.2 ± 1.3 weeks	9 eyes	6 eyes	26.4 ± 1.8 weeks	3 eyes
Group 2	6	4	24.16 ± 1.83 weeks	2 eyes	2 eyes	25.1 ± 1.3 weeks	1 eye
